# Investigation of Potential Genetic Biomarkers and Molecular Mechanism of Ulcerative Colitis Utilizing Bioinformatics Analysis

**DOI:** 10.1155/2020/4921387

**Published:** 2020-03-03

**Authors:** Jiaqi Zhang, Xue Wang, Lin Xu, Zedan Zhang, Fengyun Wang, Xudong Tang

**Affiliations:** ^1^Department of Gastroenterology, Xiyuan Hospital of China Academy of Chinese Medical Sciences, Beijing 100091, China; ^2^Experimental Research Center of China Academy of Chinese Medical Sciences, Beijing 100700, China; ^3^China Academy of Chinese Medical Sciences, Beijing 100700, China

## Abstract

**Objectives:**

To reveal the molecular mechanisms of ulcerative colitis (UC) and provide potential biomarkers for UC gene therapy.

**Methods:**

We downloaded the GSE87473 microarray dataset from the Gene Expression Omnibus (GEO) and identified the differentially expressed genes (DEGs) between UC samples and normal samples. Then, a module partition analysis was performed based on a weighted gene coexpression network analysis (WGCNA), followed by pathway and functional enrichment analyses. Furthermore, we investigated the hub genes. At last, data validation was performed to ensure the reliability of the hub genes.

**Results:**

Between the UC group and normal group, 988 DEGs were investigated. The DEGs were clustered into 5 modules using WGCNA. These DEGs were mainly enriched in functions such as the immune response, the inflammatory response, and chemotaxis, and they were mainly enriched in KEGG pathways such as the cytokine-cytokine receptor interaction, chemokine signaling pathway, and complement and coagulation cascades. The hub genes, including dual oxidase maturation factor 2 (DUOXA2), serum amyloid A (SAA) 1 and SAA2, TNFAIP3-interacting protein 3 (TNIP3), C-X-C motif chemokine (CXCL1), solute carrier family 6 member 14 (SLC6A14), and complement decay-accelerating factor (CD antigen CD55), were revealed as potential tissue biomarkers for UC diagnosis or treatment.

**Conclusions:**

This study provides supportive evidence that DUOXA2, A-SAA, TNIP3, CXCL1, SLC6A14, and CD55 might be used as potential biomarkers for tissue biopsy of UC, especially SLC6A14 and DUOXA2, which may be new targets for UC gene therapy. Moreover, the DUOX2/DUOXA2 and CXCL1/CXCR2 pathways might play an important role in the progression of UC through the chemokine signaling pathway and inflammatory response.

## 1. Introduction

Ulcerative colitis (UC) is a chronic nonspecific inflammation of the rectum and colon whose etiology and pathogenesis are not yet well defined [1]. UC has a high incidence in western countries, with increasing incidence in the developing countries [2]. The etiology of UC is considered to be multifactorial, including genetic and environmental factors such as urban lifestyles, dietary factors, high levels of hygiene, and gut microbiota, all of which are associated with disease progression; however, the pathogenesis of UC remains unclear [3]. Bioinformatics can be effectively used to analyze UC microarray data, providing theoretical reference for further exploration of the mechanisms of inflammatory bowel disease, and help to find potential target genes. As the latest bioinformatics research method, WGCNA is commonly used to reveal differences between genes in different samples [4].

In this study, UC gene expression data uploaded by Li et al. were downloaded. We identified the DEGs between UC samples and normal samples. Then, a module partition analysis was performed based on a WGCNA, followed by pathway and functional enrichment analyses. Then, data validation was performed to ensure the reliability of the hub genes. This study forecasts the molecular mechanism of UC and the potential biomarkers for UC therapy.

## 2. Materials and Methods

### 2.1. Microarray Data

The gene expression profile of GSE87473 was obtained from the GEO database [5] (http://www.ncbi.nlm.nih.gov/geo/). A total of 127 mucosal biopsy samples were obtained from 106 UC patients and 21 control subjects for subsequent analysis. The UC samples consisted of adult UC samples (*n* = 87) and pediatric UC samples (*n* = 19). Adult UC patients of 44 male and 43 female were enrolled from all geographic regions of the USA and from both metropolitan and rural settings, with an average age of 41 (race of samples not available) [6]. Pediatric UC patients of 8 male and 11 female obtained from a phase 1b clinical trial of golimumab in pediatric patients, with an average age of 15, and only subjects of European ancestry were applied [7].Normal samples (*n* = 21) were obtained from the Department of Gastroenterology, Perelman School of Medicine at the University of Pennsylvania (Philadelphia, PA) and the Department of Gastroenterology, University Hospital Gasthuisberg (Leuven, Belgium) [6], and information on age, gender, and race was not available.

### 2.2. Data Preprocessing and DEG Analysis

There were a total of 20741 probes in the present dataset. GEO2R (http://www.ncbi.nlm.nih.gov/geo/geo2r/) is based on *R* that comes with the GEO databases, which was used to identify DEGs between UC and control samples. |log‐fold change(LFC)| > 1 and *P* values <0.05 were selected as the thresholds for DEG screening.

### 2.3. WGCNA Analysis

The coexpression network analysis was performed using WGCNA (version: 1.63) [8]. WGCNA is a systematic biological method for constructing scale-free networks using gene expression data. First, we selected the soft threshold for network construction. The soft threshold was used to transform the similarity matrix of gene expression into adjacency matrix, which enhances strong correlation and weakens correlation at the exponential level. Second, the adjacency matrix was transformed into a topological matrix. Based on TOM, we used the average-linkage hierarchical clustering method to cluster genes. According to the standard of hybrid dynamic cut tree, we set the minimum number of base 30 for each gene network module. After determining the gene module by the dynamic shearing method, we calculated the eigenvectors of each module in turn, then clustered the modules, merged the nearer modules into new modules, and set height = 0.25 [9]. Third, we calculated the module eigengene (ME) of each module, which represents the expression level for each module. We also calculated the correlation between the clinical traits and ME in each module. At last, we calculated the gene significance (GS) of each gene in the module, which represented the correlation between the genes and sample.

### 2.4. Function and Pathway Enrichment Analysis

We used the DAVID 6.8 (https://david.ncifcrf.gov) software for the GO-biological function (GO-BP) and KEGG pathway analyses of the genes in main modules. We selected the P-false discovery rate (FDR) of <0.05 as the threshold for the identification of significant GO-BP terms and KEGG pathways.

### 2.5. Hub Genes Investigation

According to the feature vector of each module, the correlation of the gene expression in the module was analyzed by WGCNA. Genes with correlations greater than 0.9 in each module were considered hub genes.

### 2.6. Data Validation

To verify the robustness of hub genes, the microarray data of GSE75214 [10] ([HuGene-1_0-st] Affymetrix Human Gene 1.0 ST Array [transcript (gene) version]), which included 108 tissue samples (97UC samples and 11 control samples), were downloaded from the GEO database. GraphPad Prism 7.00 software was used to calculate the area under the curve (AUC).

## 3. Results

### 3.1. DEGs between UC Samples and Normal Samples

**Figure 1:**
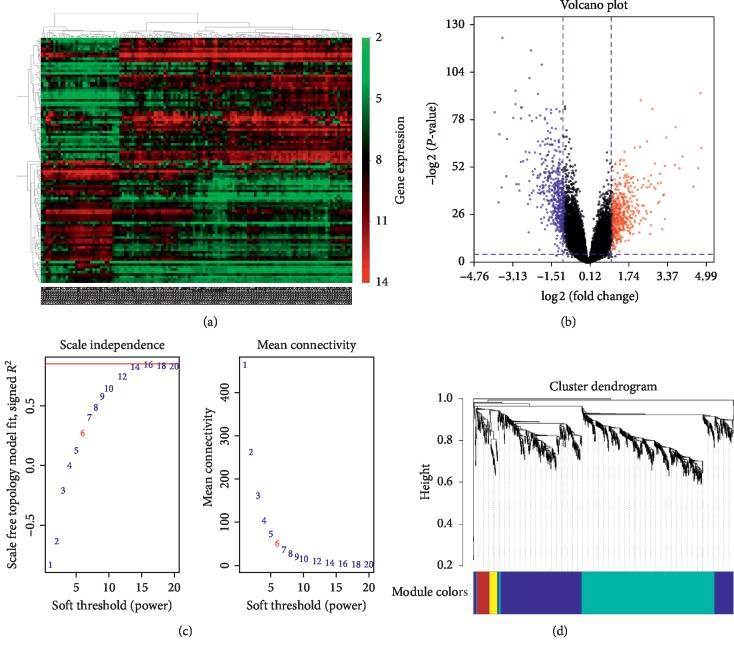
The heat map, volcano plot, and weighted gene coexpression network analysis (WGCNA) of differentially expressed genes (DEGs) between the UC group and the control group. (a) The heatmap for DEGs. (b) The volcano plot for DEGs. Gray dots represent the genes that are not differentially expressed, red dots represent the upregulated genes, and the blue dots represent the downregulated genes. (c) Determination of the soft threshold in the WGCNA algorithm. The approximate scale-free fit index can be attained at the soft-thresholding power of 6. (d) Clustering dendrograms showing 4 modules that contain a group of highly connected genes. Each designated color represents a certain gene module.

We identified 988 DEGs, including 466 upregulated DEGs and 522 downregulated DEGs with *P*_FDR_ < 0.05 and |LFC| > 1. The heatmap and volcano plot are shown in Figures 1(a) and 1(b). Obviously, the heatmap showed that these DEGs could be used to distinguish UC from control samples.

### 3.2. WGCNA Analysis

**Figure 2:**
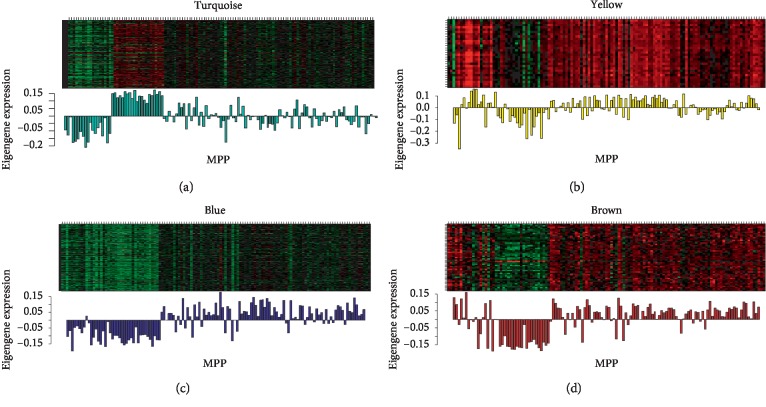
The module expression pattern. The heatmap represents the expression of genes where each row represents a gene and each column represents a sample. The red color in the heatmap represents upregulated genes, while the green color represents downregulated genes. The bar charts represent the eigengene profiles of four WGCNA modules; the color of the bar chart represents the color of the related module.

**Figure 3:**
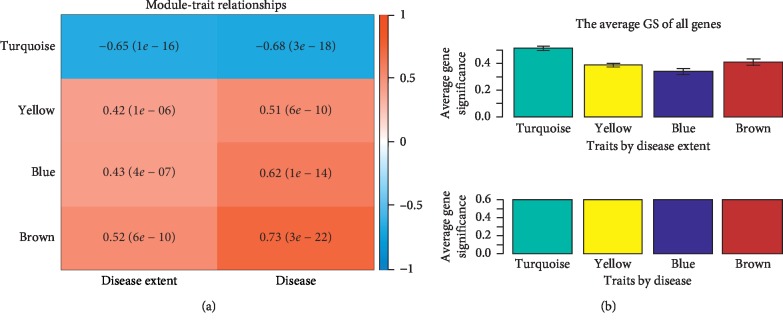
Relationships of module eigengenes and the samples. (a) The module-trait relationships. The number in the first row of the square on the right is the correlation coefficient to the UC group shown at the top of each row with the *P* values printed below the correlations in parentheses, and the number on the left is the correlation coefficient to the disease extent (limited or extensive) of the UC group. The rows are colored based on the correlation of the module to the UC group: red for a positive correlation and blue for a negative correlation. (b) The average gene significance (GS) of all genes (i.e., module significance, MS) of each module. Modules with greater MS values were considered to have more connection with the disease.

We performed WGCNA analysis using the 988 DEGs. The coexpression network is a scale-free network, which means the logarithm log(*k*) of a node with a connection degree of *k* is negatively correlated with the logarithm log(*P*(*k*)) of the probability of occurrence of the node, and the correlation coefficient is greater than 0.8. *R* software package WGCNA was used to build a weighted coexpression network. To ensure that the network was a scale-free network, we chose a soft threshold of *β* = 6 (Figure 1(c)). The DEGs were clustered into 5 modules, described here as including turquoise (510 DEGs), blue (393 DEGs), brown (48 DEGs), yellow (30 DEGs), and gray (7 DEGs) (Figure 1(d)). The turquoise and blue modules were downregulated, while the brown and yellow modules were upregulated (Figure 2). Moreover, the turquoise module (correlation index: −0.68, *P*=3.0*E* − 18) was negatively correlated with the disease presence and the yellow (correlation index: 0.51, *P*=6.0*E* − 10), blue (correlation index: 0.62, *P*=1.0*E* − 14), and brown modules (correlation index: 0.73, *P*=3*E* − 22) were positively correlated with the disease presence; while the turquoise module (correlation index: −0.65, *P*=1.0*E* − 16) was negatively correlated with the disease extent, the yellow (correlation index: 0.42, *P*=1.0*E* − 16), blue (correlation index: 0.43, *P*=4*E* − 07), and brown modules (correlation index: 0.52, *P*=6*E* − 10) were positively correlated with the disease extent (Figure 3(a)).The average gene significance (GS) for each module indicated that the brown module was the most related to disease presence, and the turquoise module was most related to disease extent (limited or extensive) (Figure 3(b)).

### 3.3. Functional and Pathway Enrichment for DEGs

**Figure 4:**
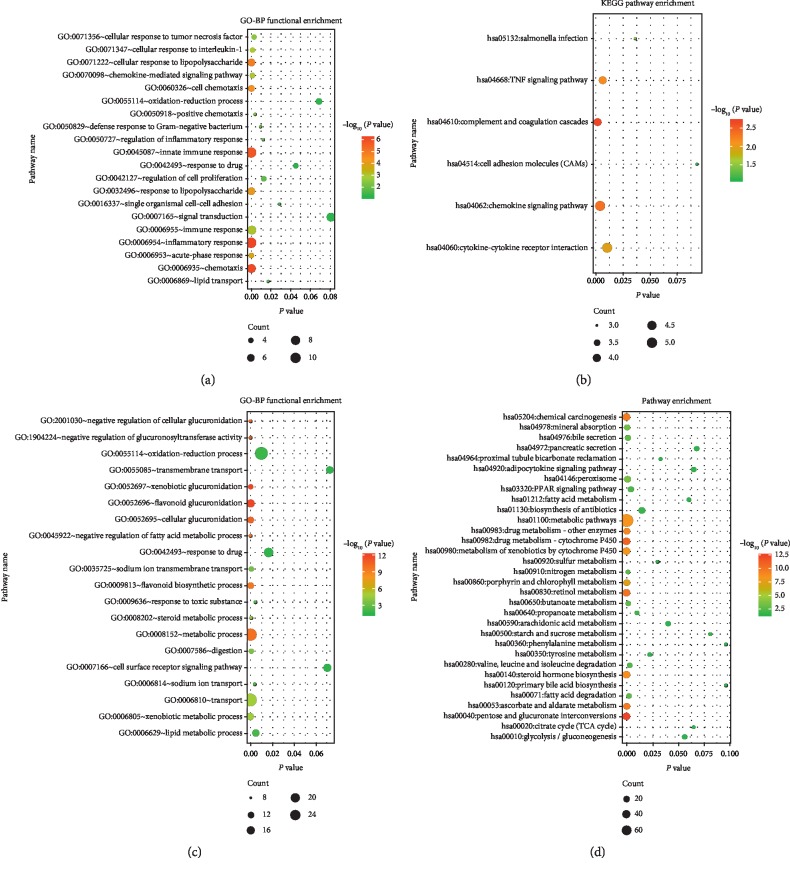
The results for GO-BP function and KEGG pathway enrichment analysis (the top 20 in the brown- and turquoise-colored modules are listed). (a) The GO-BP function enrichment of DEGs in the brown module. (b) KEGG pathway enrichment of the DEGs in the brown module. (c) The GO-BP function enrichment of the DEGs in the turquoise module. (d) KEGG pathway enrichment of the DEGs in the turquoise module.

The top 3 GO-BP and KEGG terms enriched by DEGs are shown in Table 1 and Figure 4. The DEGs in the brown module were mainly involved in functions such as inflammatory response (*P*=4.88*E* − 07) and pathways such as the chemokine signaling pathway (*P*=0.004195). The DEGs in the turquoise module were mainly involved in functions such as the oxidation-reduction process (*P*=9.70*E* − 3) and pathways such as metabolic pathways (*P*=2.8*E* − 09).

### 3.4. Hub Genes

**Figure 5:**
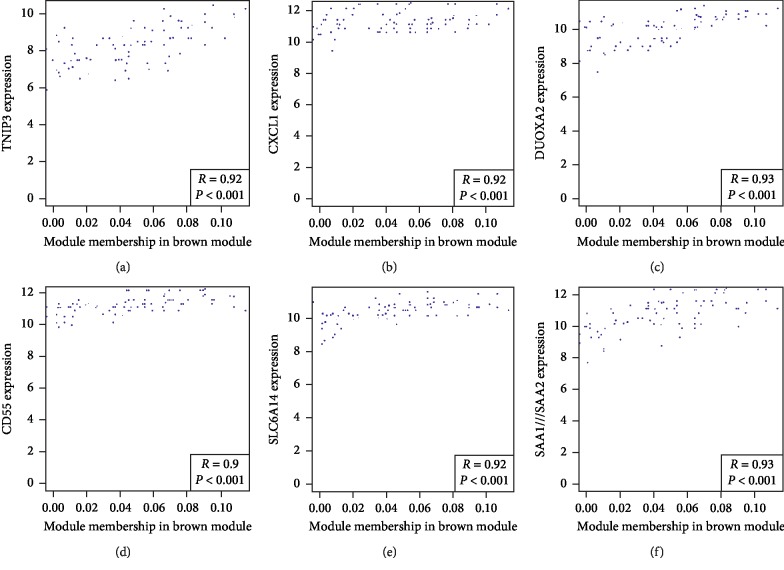
Hub genes in the brown module. A total of six genes were selected as hub genes, and the correlation coefficients (ranging from 0.90–0.93) and *P* values are shown in the lower right corner of each image.

The brown module was most relevant to the disease; therefore, we analyzed the correlation of gene expression in the brown module in the following study.  Figure 5 shows that dual oxidase maturation factor 2 (DUOXA2), serum amyloid A (SAA) 1 and SAA2, TNFAIP3-interacting protein 3 (TNIP3), C-X-C motif chemokine (CXCL1), solute carrier family 6 member 14 (SLC6A14), and complement decay-accelerating factor (CD antigen CD55) were selected as hub genes.

### 3.5. Data Validation

**Figure 6:**
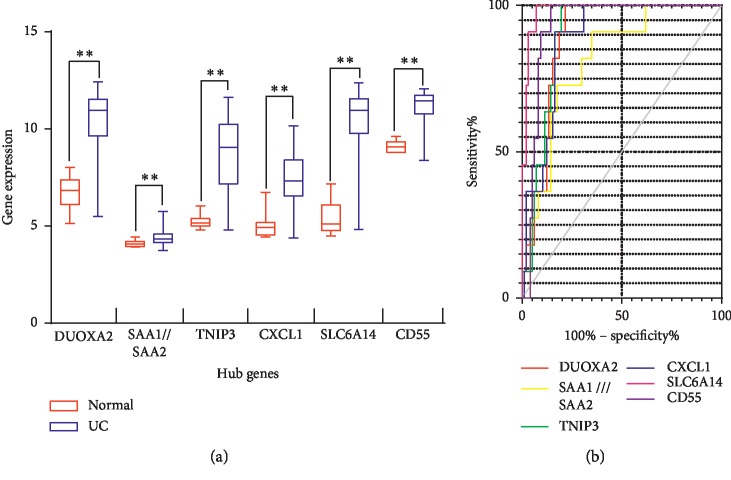
Predicted ROC curves of the UC hub genes. The prediction of UC vs. control was robust, and the area under the curve (AUC) of 6 hub genes for UC vs. control ranged from 0.8097 to 0.9822.

To verify the robustness of the hub genes, the validation data GSE75214 were obtained from the GEO database. We performed ROC curve analysis using GraphPad Prism7.00. The results of the analysis showed that the hub genes related to UC, including DUOXA2, SAA1, SAA2, TNIP3, CXCL1, SLC6A14, and CD55, were identified as potential tissue biopsy molecules for UC diagnosis (Table 2 and Figure 6).

## 4. Discussion

UC is a kind of inflammatory bowel disease that is difficult to treat, easy to recur, and prone to cancerization [11, 12].Recently, many potential biomarkers for early diagnosis or treatment of UC have been identified after the development of biology technology; however, the mechanism of UC is still unknown. In this study, UC gene expression data were analyzed by WGCNA. We screened a total of 988 DEGs between UC samples and control samples, and identified 5 modules. Based on the correlation between the modules and occurrence or development of UC, we identified 7 hub genes after data verification. Combined with previous research, SLC6A14 and DUOXA2 might be critical biomarkers for UC diagnosis.

SLC6A14 and DUOXA2 are involved in the development and carcinogenesis of UC. Multiple sequencing or microarray studies have shown that SLC6A14 was upregulated in UC patients [5, 10], 58 [13]. SLC6A14 may be involved in colonic inflammation by regulating glutamine (a substrate for SLC6A14) and nitric oxide synthase 2 (coordinated upregulation with SLC6A14 in inflamed cells) [14, 15]. Furthermore, SLC6A14 is one such cancer-specific amino acid transporter and is essential for tumor growth [16]. DUOXA2, an ROS-generating enzyme expressed in the lower gastrointestinal tract, plays a critical role in host mucosal defense [17], which could be induced by the changes of gut microbiota [18]. DUOXA2 is the maturation partner of DUOX2, which participates in the signaling pathways against inflammation and regulates reactive oxygen species (ROS), mucin, IL-8, and matrix metalloproteinase-9 against invading microbial pathogens [19]. However, overproduction of H_2_O_2_ could lead to oxidative stress resulting in oxidative injuries and mucosal barrier impairment [20]. In addition to its role in the persistent and recurrent inflammatory of UC, the DUOXA2/DUOX2 pathway is also involved in the development of UC-associated adenomas and colorectal cancer [21–23]. We supposed that SLC6A14 and DUOXA2 aberrantly expressed might promote the initiation and development of UC.

Our research also found that SAA, TNIP3, CD55, and CXCL1 were potential biomarkers for UC. SAA can reflect inflammation of UC at an early stage due to its higher sensitivity and specificity [24–26]. CXCL1 acts by specifically binding to its receptor, C-X-C chemokine receptor type 2 (CXCR2) [27]. Recent studies have shown that the CXCL1/CXCR2 signaling pathway regulates the inflammatory response; moreover, the pathway causes tumor cell proliferation, angiogenesis, and lymph angiogenesis and promotes tumor invasion and vascular metastasis [28]. Previous studies have shown increased CD55 in stools and colonic mucosa of disease activity in patients with UC [29, 30] and CD55 as the decay-accelerating factor can reflect the carcinogenesis of UC [31–33]. TNIP3 is a negative regulator of nuclear factor (NF)-*κ*B signal transduction in response to multiple stimuli [34]. Ishani Majumdar's study has demonstrated that the expression of TNIP3 negatively correlates with diseases severity in UC [35], which was contrary to our results. The contrary results might be related to the difference in disease severity and the genetic testing method.

The results of functional and pathway DEGs enrichment in this study show that the biological functions involved in the pathogenesis of UC include the inflammatory response, innate immune response, and chemotaxis, indicating that the pathogenesis of UC was multifactorial, involving epithelial barrier defects, genetic predisposition, environmental factors, and dysregulated immune responses. The 7 hub genes screened in this study are not only related to mucosal inflammation but they also accelerate the progression of colon cancer, so they should be given proper attention in the treatment of UC.

Although we found 7 hub genes closely related to UC and confirmed the robustness of their diagnostic value, which may be useful for us to improve our understanding of the molecular mechanism of UC and as a potential prognostic and diagnostic biomarker, however, there were some limitations in this study such as small sample size and lack of verification test; thus, we still need large sample size with a wide verification analysis to confirm our hypothesis.

## 5. Conclusions

In conclusion, DUOXA2, A-SAA, TNIP3, CXCL1, SLC6A14, and CD55 might be used as potential biomarkers for UC tissue biopsy, especially SLC6A14 and DUOXA2, which may be new targets for UC gene therapy. Furthermore, DUOXA2/DUOX2 and CXCL1/CXCR2 pathways may play important roles in UC progression via the inflammatory response.

## Figures and Tables

**Table 1:** The results for GO-BP function and KEGG pathway enrichment analysis (top 3 in the brown and turquoise module are listed).

Module	GO-BP terms	*P* value	KEGG terms	*P* value
Brown	Inflammatory response	4.88*E* − 07	Chemokine signaling pathway	0.004195
	Innate immune response	1.39*E* − 06	Cytokine-cytokine receptor interaction	0.010708
	Chemotaxis	7.32*E* − 07	Complement and coagulation cascades	0.001897

Turquoise	Oxidation-reduction process	9.7*E* − 3	Metabolic pathways	2.8*E* − 09
	Transport	1.4*E* − 5	Drug metabolism, cytochrome P450	1.3*E* − 11
	Metabolic process	1.2*E* − 10	Chemical carcinogenesis	2.1*E* − 10

**Table 2:** Results of AUCs for hub genes.

Hub genes	UC vs. normal		
	AUC	*P* value	95% CI
Disease-related			
DUOXA2	0.8894	<0.0001	0.826 to 0.9528
SAA1///SAA2	0.8097	0.0008	0.6975 to 0.9220
TNIP3	0.8969	0.0002	0.8366 to 0.9572
CXCL1	0.8857	<0.0001	0.8151 to 0.9562
SLC6A14	0.9822	<0.0001	0.9606 to 1.004
CD55	0.9297	<0.0001	0.8814 to 0.978

## Data Availability

The datasets analyzed during the current study are available in the GeneExpression Omnibus with the accession GSE87473 and GSE75214.

## Abbreviations

AS: Ankylosing spondylitis
AUC: Area under the curve
CD: Complement decay-accelerating factor
CXCL1: C-X-C motif chemokine
CXCR2: C-X-C chemokine receptor type 2
DEG: Differentially expressed gene
DUOXA2: Dual oxidase maturation factor 2
FDR: False discovery rate
GEO: Gene expression omnibus
GO-BP: GO-biological function
GS: Gene significance
IBD: Inflammatory bowel disease
LFC: Log-fold change
ME: Module eigengene
NF: Nuclear factor
ROS: Reactive oxygen species
SAA: Serum amyloid A
SLC6A14: Solute carrier family 6 member 14
TNIP3: TNFAIP3-interacting protein 3
UC: Ulcerative colitis
WGCNA: Weighted gene coexpression network analysis.

## Supplementary Materials

Supplementary Materials The supplementary materials provide the specific URL of the dataset used in the manuscript.
