# Supplementary material for: Investigation of Potential Genetic Biomarkers and Molecular Mechanism of Ulcerative Colitis Utilizing Bioinformatics Analysis
Source: Biomed Res Int. 2020 Mar 3;2020:4921387. doi: 10.1155/2020/4921387 (PMC7073481; doi:10.1155/2020/4921387)
Supplement: Supplementary Materials — The supplementary materials provide the specific URL of the dataset used in the manuscript. [file 4921387.f1.docx]

**Supplementary information:** Our dataset is available on the following web page: <https://www.ncbi.nlm.nih.gov/geo/query/acc.cgi?acc=GSE87473> and <https://www.ncbi.nlm.nih.gov/geo/query/acc.cgi?acc=GSE75214>
